# Atypical Presentation of Sjögren-Larsson Syndrome

**DOI:** 10.1155/2017/7981750

**Published:** 2017-10-18

**Authors:** D. Papathemeli, A. Mataftsi, A. Patsatsi, D. Sotiriadis, M. Samouilidou, S. Chondromatidou, A. Evangeliou

**Affiliations:** ^1^2nd Department of Dermatology and Venereology, Faculty of Medicine, Aristotle University of Thessaloniki, Papageorgiou General Hospital, Thessaloniki, Greece; ^2^2nd Department of Ophthalmology, Faculty of Medicine, Aristotle University of Thessaloniki, Papageorgiou General Hospital, Thessaloniki, Greece; ^3^Department of Radiology, Papageorgiou General Hospital, Thessaloniki, Greece; ^4^4th Department of Pediatrics, Faculty of Medicine, Aristotle University of Thessaloniki, Papageorgiou General Hospital, Thessaloniki, Greece

## Abstract

Sjögren-Larsson syndrome is a rare neurocutaneous disorder characterized by ichthyosis, spastic diplegia or tetraplegia, and intellectual disability. Herein, we describe a case of a Greek patient with ichthyosis and spasticity of the legs but with normal intelligence (IQ 95). This syndrome should be suspected when a child presents with ichthyosis and spastic diplegia or tetraplegia, even if intelligence is normal.

## 1. Introduction

Sjögren-Larsson syndrome (SLS) is a rare neurocutaneous disorder caused by deficient activity of fatty aldehyde dehydrogenase (FALDH) and is characterized by congenital ichthyosis, spastic diplegia or tetraplegia, and intellectual disability. This is a case of SLS with atypical presentation, expanding the phenotypic spectrum of the syndrome.

## 2. Case Presentation

A 3-year-old girl of Greek descent was referred to our hospital due to gait disturbance and undiagnosed skin lesions.

**Figure 1:**
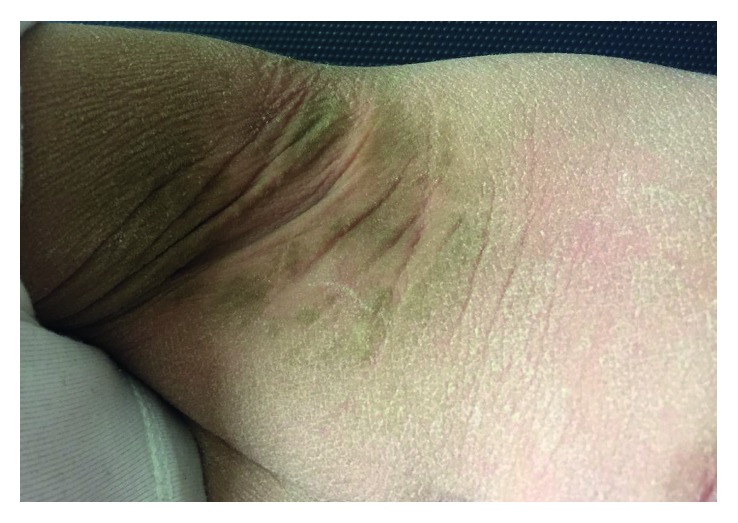
The patient presented with thickening and hyperpigmentation of both axillae.

The patient presented bilateral spasticity of the legs. Acquisition of motor skills was delayed, as she achieved standing without support and walking at the age of 13 and 16 months, respectively. At the age of 16 months, she spoke 8–10 words. There was no history of epileptic seizures. Upon examination, she presented with extensive hyperkeratosis and scaling of the skin especially on palms and soles and hyperpigmented skin flexures (Figures 1()–3()). Her hair and fingernails were normal. There was a history of recurrent chalazia. Her birth was at full-term (39 weeks), following an uneventful pregnancy. She is the fourth living child of a consanguineous couple who reported being third cousins, all other siblings having no significant medical history.

Height (104 cm), weight (16.5 kg), and head circumference (53.5 cm) were within normal age limits at presentation. Neurological evaluation revealed spastic diplegia of lower extremities, mild generalized increase in muscle tone, deep tendon reflexes of the lower extremities, and positive Babinski reflexes bilaterally. Intelligence was normal (IQ 95). Electroencephalopathy, otoacoustic emission test (OAE), abdominal and renal ultrasound, and X-ray of the chest and hips appeared to be normal.

**Figure 4:**
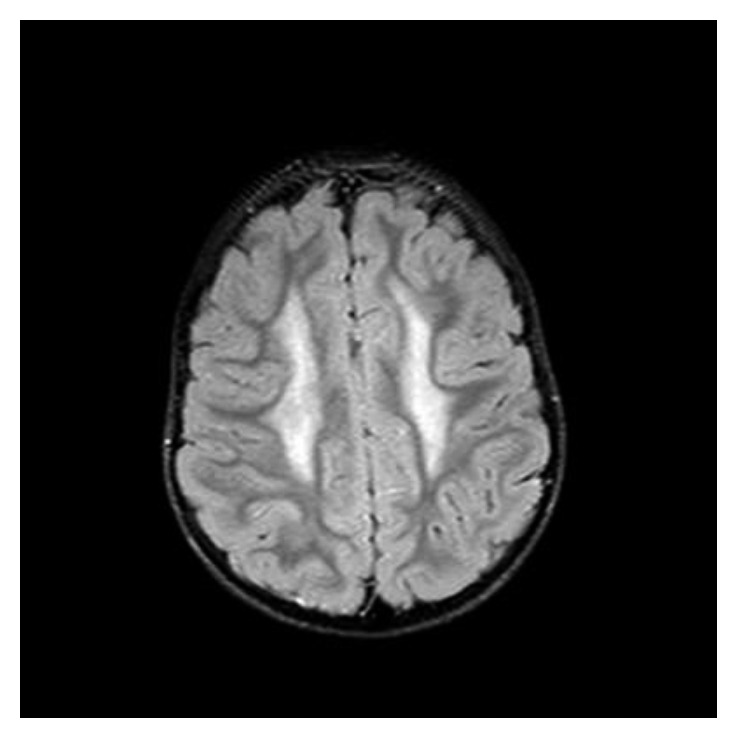
The deep white matter of the centrum semiovale bilateral demonstrates high signal intensity changes at Flair axial MR imaging.

Conventional magnetic resonance imaging (MRI) of the brain disclosed diffuse symmetrical high-intensity lesions in the deep white matter of the centrum semiovale and the frontal lobes and milder-intensity diffuse lesions in the posterior parietal lobes and the corpus callosum, while magnetic resonance spectroscopy (MRS) showed moderate increase of lipid and myoinositol levels. Other metabolites like *N*-acetyl aspartate (NAA), choline (Cho), and creatine (Cr) were within normal limits (Figures 4()–6()).

Fundoscopy was performed to search for signs of metabolic retinopathy, and no abnormal features were detected in either eye. Absence of retinal deposits or other pathology was confirmed with optical coherence tomography of the macula. The patient had a history of recurrent chalazia. Cycloplegic retinoscopy revealed high refractive error (right eye C −4.50 D × 20°, left eye C −4.00 D × 170°). Visual acuity, measured with the ETDRS chart at 4 meters, was LogMAR 0.5 in the right eye and LogMAR 0.3 in the left eye with her glasses, which was presumed to be the result of refractive amblyopia and not of retinal pathology.

Histopathology of skin biopsy from the right axillary region and right thigh showed orthokeratotic hyperkeratosis, acanthosis, and papillomatosis of the stratum spinosum.

Clinical findings led to a differential diagnosis of Sjögren-Larsson syndrome, Refsum disease, Rud syndrome, and Chanarin-Dorfman syndrome, and so genetic testing was performed. Sequencing of the polymerase chain reaction product using the exon-specific primers revealed a c.551C>T mutation of the *ALDH3A2* gene on chromosome *17p11.2* in homozygous state and confirmed the diagnosis of Sjögren-Larsson syndrome. Parents were tested and both were found to be heterozygous for this mutation. The patient was prescribed emollient baths and moisturizing creams, while physiotherapy was advised to improve spasticity.

No other clinical signs developed during three years of follow-up.

## 3. Discussion

Sjögren-Larsson syndrome is a rare autosomal recessive hereditary neurocutaneous disorder with a worldwide incidence of 0.4 per 100,000 people and was first described by Sjögren and Larsson in 1957 [1]. It is caused by deficient activity of fatty aldehyde dehydrogenase (FALDH), a component of the fatty alcohol nicotinamide adenine dinucleotide (NAD) oxidoreductase enzyme complex (FAO). FALDH deficiency results in the accumulation of fatty aldehydes and fatty alcohols in body tissues, which causes the symptoms [2]. This syndrome is characterized by the triad of congenital ichthyosis, spastic diplegia or tetraplegia, and mild to moderate intellectual disability (most patients show an intellectual coefficient of less than 50). Less common features are preterm birth, reduced visual acuity and photophobia, short stature, kyphoskoliosis, seizures, and delayed speech (spastic dysarthria may also be present) [3, 4].

FALDH deficiency affects epidermal functioning, and leaky water barrier causes ichthyosis. Cutaneous features are usually congenital or apparent during the neonatal period and become more pronounced overtime, as in our case [2, 3].

Neurological symptoms emerge by early childhood (first or second year of life). Later in adolescence, the neurological clinical picture stabilizes [4, 5]. In our case, it is remarkable that the patient had normal intelligence (IQ 95).

Crystalline juvenile macular dystrophy, cystoid foveal atrophy, and lack of macular pigment are ocular features regarded as pathognomonic of Sjögren-Larsson syndrome but were not detected in our patient [6]. On the other hand, high refractive error and recurrent chalazia associated with meibomian gland dysfunction are signs that have not as yet been associated with the syndrome.

Diagnosis of SLS should be considered in children with simultaneous presence of congenital ichthyosis and debilitating neurological symptoms. However, the phenotype of SLS with the classical neurocutaneous features follows a typical age-dependent pattern and the full-blown phenotype becomes apparent after 2-3 years; therefore, SLS is usually not suspected until that age [3–5]. In our case, diagnosis was made at the age of 3 years.

Diagnosis of Sjögren-Larsson syndrome is confirmed by measurement of FALDH or fatty alcohol: NAD oxidoreductase in cultured skin fibroblasts [7] and/or sequence analysis of *ALDH3A2* gene on the locus *17p11.2.ALDH3A2* gene mutation tests are highly sensitive, do not require skin biopsy, and can complement or even replace FALDH enzymatic assays in SLS [8].

*FALDH* gene (also known as *ALDH3A2*) which encodes fatty aldehyde dehydrogenase is located on chromosome *17p11.2* and consists of 11 exons. More than 90 different mutations (amino acid substitutions, deletions, insertions, and splicing errors) have been found in patients with SLS. Missense mutations account for 38% of the known mutations in *ALDH3A2* and are scattered throughout the gene. Two mutations, c.1297-1298delGA and c.943C>T, are considered to be the most frequent in Europe [8]. In our patient, the missense mutation c.551C>T was detected, which is responsible for replacement of threonine 184 by methionine (p.T184M) at the amino acid level of the FALDH enzyme. This mutation was first described by Rizzo et al. in 1999 as pathogenic and has been detected in Europe and Middle East [9]. It has been reported that the severity of clinical phenotype does not closely correlate with specific mutations. Even among siblings who share the same genotype, phenotype can vary significantly, due to genetic and environmental modifiers [10].

As with most inherited metabolic diseases, there is no curative therapy, and treatment is predominantly symptomatic. In our patient, ichthyosis improved significantly with abundant hydration with emollient baths, moisturizing creams, and keratolytic agents. In our case, prescription of acitretin was avoided because of the risk of premature epiphyseal closure. Physiotherapy improved ambulation and prevented contracture development. Studies have shown that zileuton, a 5-lipoxygenase inhibitor, has a potent advantageous effect upon pruritus in SLS patients, since it inhibits leukotriene formation [5, 11]. Future therapeutic strategies may include bezafibrates, carotenoids, and gene therapy [4, 5]. However, further research in this field is required.

## Figures and Tables

**Figure 2:**
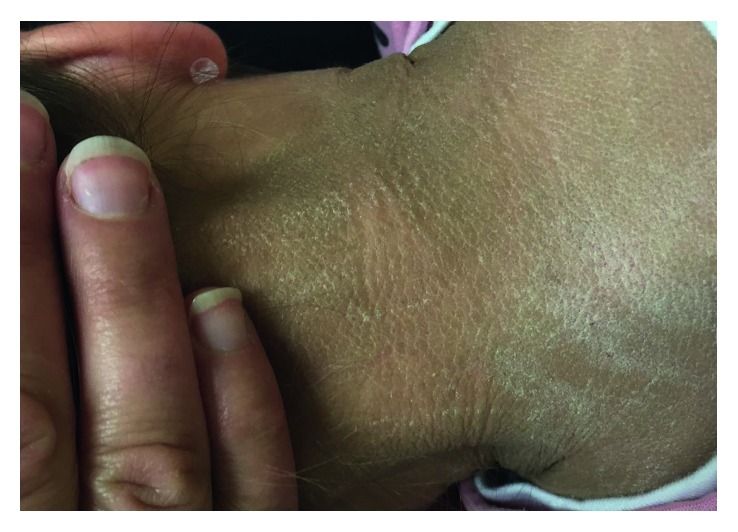
Brown scaly hyperpigmentation could also be seen on the nape of the neck.

**Figure 3:**
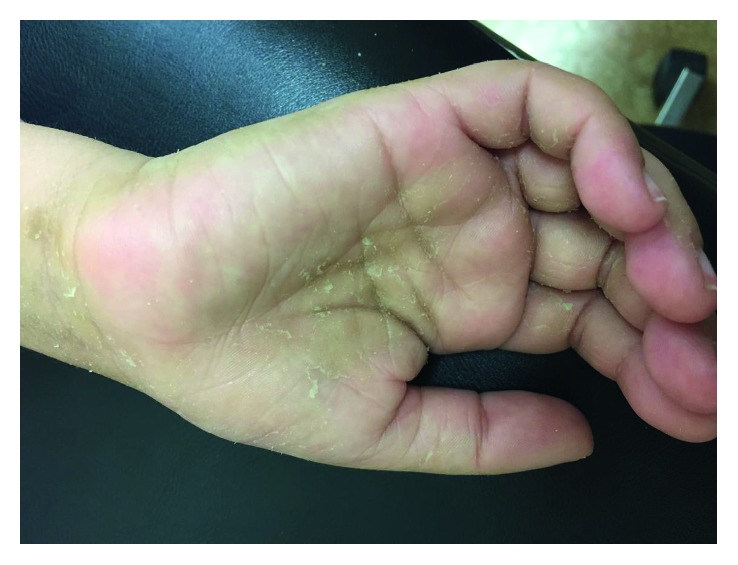
Palmoplantar keratoderma was also present.

**Figure 5:**
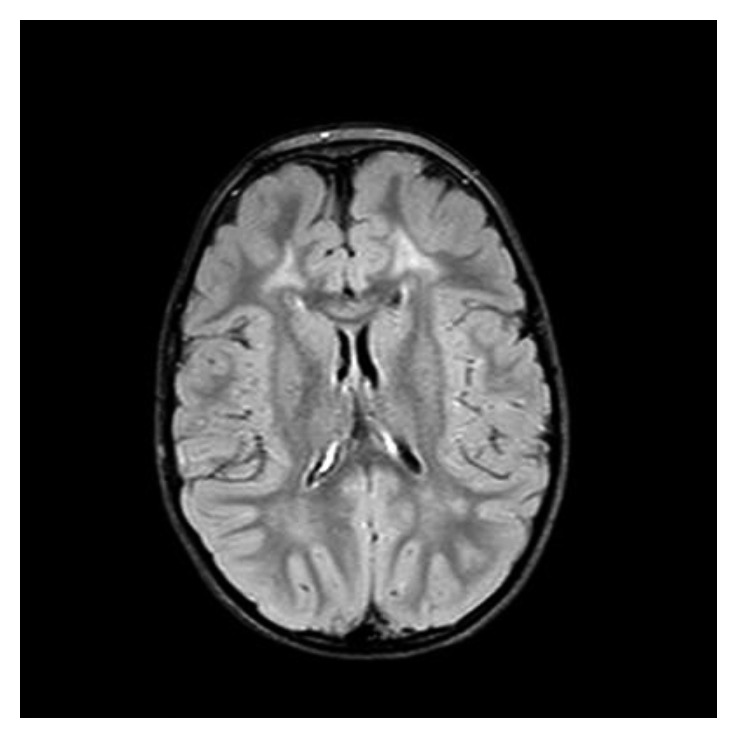
Flair axial MR image of the brain showing high-intensity lesions in the deep white matter of the frontal lobes and milder-intensity lesions in the deep white matter of the posterior parietal lobes and the corpus callosum.

**Figure 6:**
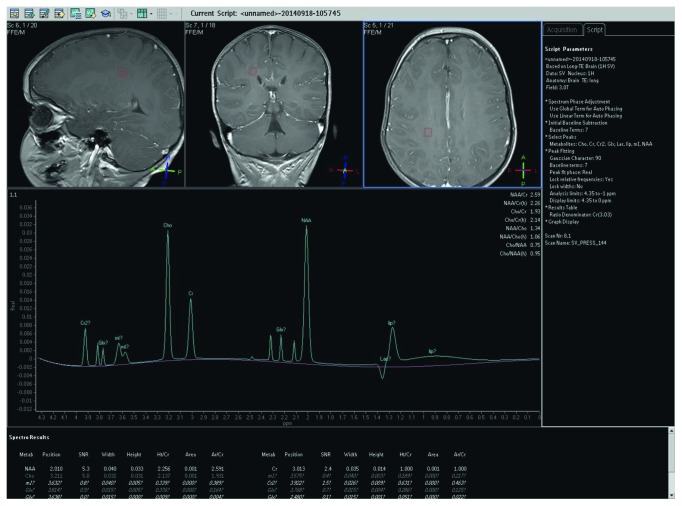
Magnetic resonance spectroscopy (MRS) showed moderate increase of lipid and myoinositol levels. Other metabolites like *N*-acetyl aspartate (NAA), choline (Cho), and creatine (Cr) were within normal limits.
